# Divergent ecological histories of two sister Antarctic krill species led to contrasted patterns of genetic diversity in their heat‐shock protein (*hsp70*) arsenal

**DOI:** 10.1002/ece3.1989

**Published:** 2016-02-09

**Authors:** Claire Papot, Kévin Cascella, Jean‐Yves Toullec, Didier Jollivet

**Affiliations:** ^1^ Université de Lille1CNRS UMR8198Groupe ‘Ecoimmunology of Marine Annelids’Bât SN2, 1er étageporte 11359655Villeneuve d'AscqFrance; ^2^ CNRSUMR 7144Equipe ABICEStation Biologique de Roscoff29682RoscoffFrance; ^3^ Laboratoire ‘Adaptation et Diversité en Milieu Marin’UPMCStation Biologique29682RoscoffFrance

**Keywords:** Balancing selection, duplication, heat‐shock proteins, krill, sweep, thermal adaptation

## Abstract

The Arctic and the Antarctic Peninsula are currently experiencing some of the most rapid rates of ocean warming on the planet. This raises the question of how the initial adaptation to extreme cold temperatures was put in place and whether or not directional selection has led to the loss of genetic variation at key adaptive systems, and thus polar species’ (re)adaptability to higher temperatures. In the Southern Ocean, krill represents the most abundant fauna and is a critical member at the base of the Antarctic food web. To better understand the role of selection in shaping current patterns of polymorphisms, we examined genetic diversity of the *cox‐1* and *hsp70* genes by comparing two closely related species of Euphausiid that differ in ecology. Results on mt*cox‐1* agreed with previous studies, indicating high and similar effective population sizes. However, a coalescent‐based approach on *hsp70* genes highlighted the role of positive selection and past demographic changes in their recent evolution. Firstly, some form of balancing selection was acting on the inducible isoform C, which reflected the maintenance of an ancestral adaptive polymorphism in both species. Secondly, *E. crystallorophias* seems to have lost most of its *hsp70* diversity because of a population crash and/or directional selection to cold. Nonsynonymous diversities were always greater in *E. superba,* suggesting that it might have evolved under more heterogeneous conditions. This can be linked to species’ ecology with *E. superba* living in more variable pelagic conditions, while *E. crystallorophias* is strictly associated with continental shelves and sea ice.

## Introduction

According to Jackson and Overpeck (2000), fitness decline in the face of global warming may be counterbalanced by being physiologically more plastic, moving to upper latitudes or evolving. In most cases, colonization of new habitats should represent one of the most appropriate solutions (Parmesan et al. 2005) and population contraction in refugial areas followed by rapid re‐expansion has been widely documented during the last glacial episodes (Hewitt 2004). However, escaping global warming is not always possible for species inhabiting restricted or closed habitats such as lakes, islands, or the polar zones. In these cases, species will have to rapidly adapt to the changing environment either by taking advantage of their inherent phenotype plasticity or putative favorable mutations associated with their standing variation. This is especially the case of Antarctic organisms, which, while living on the edge of the thermal gradient, are physiologically less capable of enduring thermal changes (Peck et al. 2014).

The Antarctic fauna have evolved for millions years in a very constrained environment with subzero temperatures driving peculiar adaptations (Rogers et al. 2012). The ecological isolation of the fauna with the progressive cooling of the continent indeed started about 35 My ago (Miller et al. 1991) leading to a relatively stable and cold environment during the last 5 My, within which many glacial cycles have been documented (Naish et al. 2009). As a consequence, evolution in the persistently cold and oxygen‐rich Antarctic waters has thus forged molecular and genomic changes leading to physiological and biochemical adaptation. First of all, the long‐term adaptation to low temperatures has been well recognized to influence both the structural conformation and the performance of proteins. In many polar species, proteins have evolved toward more flexibility in order to maintain their optimal functional state (D'Amico et al. 2002; Siddiqui and Cavicchioli 2006). More specifically, species endured strong directional selection leading to the loss of some genes (i.e., loss of ability to express hemoglobin and myoglobin proteins for fishes of the family Channichthyidae (Cocca et al. 1997; Somero et al. 1998; Sidell and O'Brien 2006)) or the emergence of novel physiological functions (i.e., gain of antifreeze glycoproteins in subzero, ice‐filled waters (DeVries 1988; Chen et al. 1997)). Among them, diversification and ability of heat‐shock proteins to respond to temperature challenges may have also played a role in the evolution of the polar fauna. Heat‐shock proteins are indeed well known to improve the short‐term plasticity of the physiological response of an organism to elevated temperatures (Buckley et al. 2001) but have also been reported to be sensitive to cold, both in terms of gene expression and allelic frequencies along altitudinal (Dahlhoff and Rank 2007) and latitudinal (Hoffmann et al. 2002; Weeks et al. 2002) clines. In Antarctic metazoans, the uniform constraint of cold conditions over millions of years possibly led to the loss of inducibility of the *hsp* expression (Feder and Hofmann 1999; Hofmann et al. 2000; Buckley et al. 2004; Place et al. 2004; Clark and Peck 2009) or non‐negligible geographic variation in their level of expression from North to South (Barbosa et al. 2007).

One main consequence of long‐term adaptation to extreme but stable thermal conditions is the reduction of genetic diversity by positive directional selection which promotes selective sweeps and genetic hitchhiking at neutral linked sites (Maynard Smith and Haigh 1974; Fay and Wu 2000; Przeworski et al. 2005). But, genetic diversity reduction may be also reinforced by the past demographic history of the polar species. Climatic oscillations of the Quaternary seem to have indeed played a non‐negligible role in shaping most of the observed patterns of genetic variation in terrestrial and marine species with accentuated effects in species presently living at the highest latitudes (Hewitt 2004; Sromek et al. 2015). During glacial periods, benthic organisms of Antarctica have been heavily impacted with a strong reduction in their effective population size, resulting in bottlenecks (Rogers et al. 2012). When comparing species with similar life‐history traits, those inhabiting polar regions should therefore display more limited evolutionary responses to changing conditions due to their putative lower standing variation (Amos and Balmford 2001) as the direct consequence of their past demographic history (Booy et al. 2000; Willi and Hoffmann 2009). In this respect, comparing the amount of genetic variation of key “adaptive” genes between closely related species sharing the same ecological niche but different ecological constraints and population history should provide valuable information about their putative adaptability and the relative role of selection and past demography to this extent.

The emblematic Antarctic krill *Euphausia superba* and its sister species *Euphausia crystallorophias,* also called “ice krill”, represent keystone species of the Southern Ocean food chain (Trivelpiece et al. 2011; Hill et al. 2013). Both species have a circumpolar distribution, forming large swarms over the Antarctic waters in the summer period. However, their geographic range does not overlap and distributions are likely governed by subtle ecological preferences. The Antarctic krill *E. superba* (Eus) lives in open waters of the austral ocean but is not observed up to 74°S of latitude. It encounters a wide range of environmental conditions throughout the subaustral waters (Atkinson et al. 2004; Clarke and Tyler 2008) with vertical migration at abyssal depths where it was found to feed and reproduce actively (Clarke and Tyler 2008). While spending most of its life near to the Antarctic Circumpolar Current of the Southern Ocean (ACC) in the upper 200‐m layer (Siegel 2005), it can easily cope with water temperatures up to 3.9°C. It is therefore known to display one of the highest effective population size with high migration rates around Antarctica (Goodall‐Copestake et al. 2010; Bortolotto et al. 2011 but see: Zane et al. 1998). In contrast, the ice krill *E. crystallorophias* (Euc) is restricted to the continental inshore waters where it represents the dominant Euphausiidae. Its genetic diversity is also rather high but may be a little bit more geographically structured according to Jarman et al. (2002). The species is mainly associated with the ice shelves (Patarnello et al. 1996) where it lives in waters below 0°C with no vertical migration. The two krill species form a monophyletic lineage that diverged by vicariance from the subaustral *Euphausia vallentini* about 19 Mya as the consequence of the formation of the circum‐Antarctic water circulation and the Antarctic Polar Frontal Zone (Patarnello et al. 1996). According to molecular clock calibrations and a substitution rate of c.a. 2.5% per million years, it has been estimated that the two species separated about 6 Mya at the end of the late Miocene cooling. This was a period known to represent a strong glacial episode (Pearson and Palmer 2000).

Because of its significant contribution to the austral ocean biomass, many studies have focused on the ecological and migratory behaviors of the Antarctic krill in order to predict their population level responses to climate change. However, very few studies have addressed this issue with reference to their thermal physiology and resistance to increasing temperatures. Recently, the transcriptomes of both the Antarctic and ice krills were described, focusing on chaperone genes and particularly on the *hsp* families and the peptidome (Clark et al. 2011; Toullec et al. 2013). A previous study carried out by Cascella et al. (2015) allowed us to identify genes involved in the multigene family of the heat‐shock protein 70s and to address their level of diversification in the Antarctic and ice krills while assessing their level of functionality when subjected to moderate thermal stresses. The two krill species displayed at least 6 distinct *hsp70* isoforms including Grp78, all of them emerging from old duplication events before the isolation of the Antarctic krill lineages, 20 Mya. However, when using d_N_/d_S_ ratios and selective models of codon substitutions, most of them exhibited variable selective patterns in the branches leading to the krill species after the speciation event. More precisely, when looking at both the molecular and expression levels, Cascella et al. (2015) reported that the *hsp70C* isoform, which displays the inducible molecular signature, may have lost its inducibility following a recent selective relaxation. Although the analyses used were robust enough to detect adaptive evolution between closely related species, the method was not refined enough to discriminate the effect of natural selection on each ecological species after the speciation event.

The aim of the study was therefore to explore the selective mechanisms by which the two krill species may have differentially adapted to cold conditions by performing a polymorphism‐to‐divergence analysis of genes leading to both the constitutive and inducible forms of *hsp70*. However, the climatic history of both species may have also played a role in shaping their level of genetic diversity. Because the ice krill is restricted to more constant and extreme cold conditions while developing under the ice sheet over the continental plateau, one can expect that it should have lost some of its diversity via directional selection or stronger demographic events. Conversely, although developing under sea ice during winter, the emblematic Antarctic krill *E. superba* may have been genetically less impacted by climatic oscillations because of its wider distribution over the Austral Ocean where it encounters a larger panel of conditions during night and day. This hypothesis was therefore tested by the joint coalescence analysis of three distinct genes encoding the cytosolic Hsp70s and the mitochondrial *cox1* gene. This latter gene was included as a “neutral” reference (no nonsynonymous changes) to which the putative adaptive *hsp* loci were compared following the hypothesis that a strong reduction of genetic diversity in one species due to a recent demographic change will be first detectable at this locus.

## Material and Methods

### Sampling

**Figure 1:**
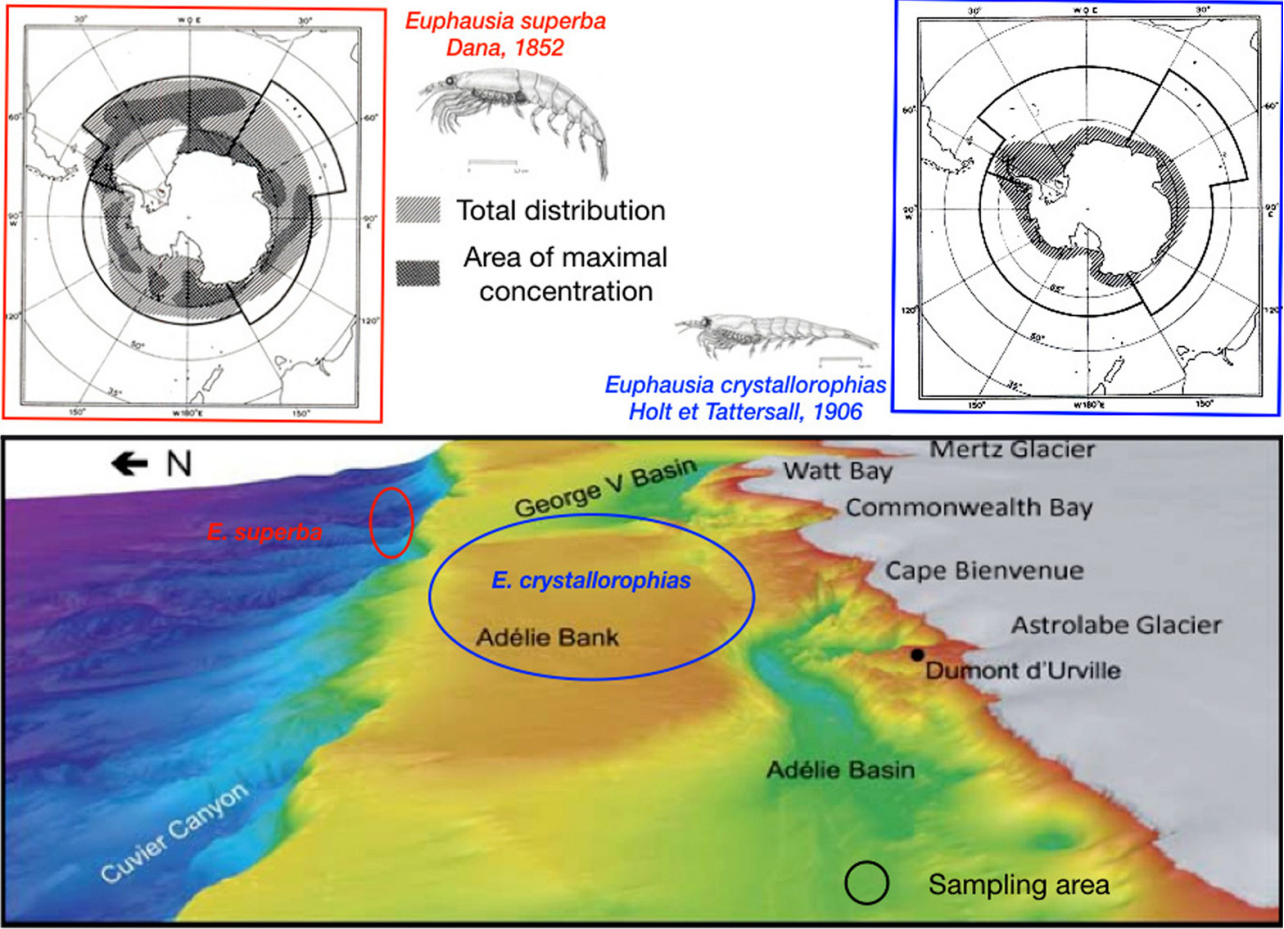
Distribution of *E. superba* and *E. crystallorophias* along the Antarctic coastline together with their sampling location at the Antarctic French base Dumont d'Urville (distribution maps were redrawn and modified after Fischer and Hureau 1987, the red to blue color gradient of the Dumont d'Urville map correspond to bathymetry from 500 m (Adelie Bank) to about 4000 m (the abyssal plain)).

Samples were collected on board of the RV *Astrolabe* using IKT‐1000 plankton net during the Summer oceanographic cruise in 2011 as part as the research project KREVET (2009–2012, coord. J.Y. Toullec). Collections were performed in the Dumont d'Urville Sea on the continental slope for *E. superba* and on the continental shelf for *E. crystallorophias* (Fig. 1). Krill swarms were always monospecific and located using an echo sounder to be fished and directly transferred into 1‐m^3^ conical tanks of open chilled (−0.5°C) and well‐oxygenated seawater. For both species, the genetic sample was obtained from a single catch (one swarm). Several hundreds of individuals were collected in one catch and used for various thermal experiments, some of which (a hundred) were kept for population genetics purposes. Animals were then measured and sexed on board using a binocular microscope and immediately frozen in liquid nitrogen or preserved in 80% alcohol for further genetic and physiological analyses.

### DNA extraction

A series of 48 individuals per species were used for DNA extraction and subsequent PCR amplification at the 4 loci. DNA was extracted from the abdominal muscle using a Qiagen DNAeasy Tissue kit according to the manufacturer's instruction for frozen animals, and using the CTAB‐1%PVP protocol adapted from Doyle and Doyle (1987) for alcohol‐conserved animals (see Jolly et al. 2003). Total genomic DNA quantity was estimated using the NanoDrop 1000 (Thermo Fisher Scientific, Asnières, France) spectrophotometer and visualized on a 1.5% TBE agarose gel following electrophoresis.

### PCR analysis

Complete *hsp70* gene sequences were obtained from either a 454 pyrosequencing for *E. superba* (Clark et al. 2011) or a Miseq Illumina run for *E. crystallorophias* (Toullec et al. 2013; Cascella et al. 2015). These sequences allowed us to design species‐specific primers from each 5′ CDS of the three paralogous genes for which no intron was recorded (see Table 1). A fragment corresponding to about half of the CDS encoding the cytosolic constitutive and inducible isoforms of *hsp70*, namely *hsp70A*,* hsp70B,* and *hsp70C* (later called Paralogs A, B, and C) was then amplified by PCR. A fragment of the mitochondrial *cox‐1* gene was also amplified using primers previously published in Goodall‐Copestake et al. 2010. DNA was amplified in a final volume of 25 *μ*L containing 2.5 *μ*L of PCR buffer (10X), 0.2% of milk, MgCl2 (2.0 mM), dNTP (0.4 *μ*mol/L), 0.2 *μ*mol/L of each primer, and 0.04U of Uptitherm Taq (Invitrogen). Amplifications were carried out for the 3 *hsp70* genes with the following conditions: a denaturation step at 94°C/3 min, a first cycle of 94°C/30 sec, 60°C/2 min, and 72°C/3 min followed by 35 cycles of 94°C/30 sec, 62°C/30 sec, and 72°C/1 min, and a final elongation step at 72°C/7 min. In the specific case of *cox‐1*, amplification conditions were as follows: 94°C/4 min followed by 35 cycles: 94°C/45 sec, 55°C/45 sec, and 72°C/1 min with a final elongation step 72°C/7 min.

**Table 1:** Species‐specific primer sequences used to amplify alleles of the first half of both of the 3 cytosolic *hsp70*‐encoded genes and the mitochondrial *cox‐1* gene

Locus	Species	Direction	5′ Primer 3′
Paralog A	*Euc*	Forward	CCATCATATGTTGCCTTCACTG
Paralog A	*Euc*	Reverse	AGTTTCAATACCCATAGAGAGAGG
Paralog A	*Eus*	Forward	CCATCATATGTTGCCTTCACAG
Paralog A	*Eus*	Reverse	AGTTTCAATACCCATAGAAAGAGG
Paralog B	Both	Forward	TGCAAATGACCAGGGTAACC
Paralog B	Both	Reverse	GCCTCCAACCAAGACGAT
Paralog C	Both	Forward	CCAAGATGTCTGCTCCAGT
Paralog C	*Euc*	Reverse	CTTGTGAAGATGAGGATAGGGT
Paralog C	*Eus*	Reverse	CTTGTGACGATGAGGATAGGGT
*cox‐1*	*Euc*	Forward	AGAATGAGGTATTCAAGTTACGG
*cox‐1*	*Euc*	Reverse	GATATTGGCACACTATACTTCAT
*cox‐1*	*Eus*	Forward	AGAATGAGGTATTTAAATTACGA
*cox‐1*	*Eus*	Reverse	GATATTGGTACATTATACTTTAT
Puc19	BS1F	Forward	AGGGGGATGTGCTGCAAGGCG
Puc19	BS1R	Reverse	CTTCCGGCTCGTATGTTGTGTG
Sequencing	SP6	Forward	CATTTAGGTGACACTATAG
Sequencing	T7	Reverse	GTAATACGACTCACTATA

### DNA sequencing

Here, a double approach using direct sequencing and the mark–recapture (MR) cloning protocol described by Bierne et al. 2007 was used taking advantage of the lack of introns in the sequenced markers. Because of the recapture method, this approach was only performed on 24 individuals from each species. The MR method involves the tagging of each individual using 4‐nucleotide sequences added to the 5′ end of the initially designed primers. Each individual was separately amplified with a couple of tagged primers and PCR products were then pooled, refreshed by adding dATP and purified following electrophoresis with the QIAEX II Purification Kit (QIAGEN, Courtaboeuf, France). The purified PCR products were subsequently cloned following a ligation in a pGEM‐T vector (pGEMT‐T cloning kit; Promega, Charbonnières‐les‐bains, France). Clones were sequenced both strands on ABI 3100 using BigDye (PerkinElmer, Courtaboeuf, France) terminator chemistry following the manufacturer's protocol, and sequences were then identified back to each individual using their pairs of tags. A 2‐time recapture effort was used in order to recover at least one allele for a maximum of individuals. In parallel, each individual was also directly sequenced with the standard primers and the second allele was deduced from the presence of double peaks (heterozygous sites) in the latter sequence when compared to the cloned one for all the recaptured individuals. Sequences of *cox‐1* were also obtained by direct sequencing and compared to the pre‐existing *cox‐1* sequences in GenBank. Sequences were finally aligned and corrected manually with the GENEIOUS software (Drummond et al. 2011).

### Genotyping

In order to test whether some allelic lineages represent putative paralogs, individuals were genotyped at the *hsp70* paralog B using a restriction fragment length polymorphism (RFLP) approach and the restriction enzyme *Aci I* targeting the deletion of the glycine codon between allelic lineages of *E. superba*. Five *μ*L of PCR product obtained from each of 48 *E. superba* specimens was incubated with the enzyme and 1% BSA during 1 h 30 at 37°C with a denaturation step of 20 min at 65°C following the manufacturer's protocol. The same individuals were also genotyped at the *hsp70* paralog C by the direct sequencing of a smaller fragment together with 24 *E. crystallorophias* to discriminate allelic lineages by checking the homo/heterozygote state of five diagnostic sites previously depicted from the alignment of alleles obtained by the MR method. The finding of at least two homozygotes allowed us to rule out the co‐occurrence of duplicated genes.

### Genetic diversity, species divergence, and statistical tests

For each gene, the number of segregating sites (S), the gene diversity (Hd), the theta of Watterson *θ*_w,_ and the nucleotide diversity *π* were estimated using DNAsp v5.10 software (Librado and Rozas 2009). The estimators *θ*_w_ and *π* (both estimating the population parameter 4N_e_.*μ*) were then compared using the Tajima's D test, D being supposed to be close to zero when the locus is evolving neutrally (i.e., no selection and no demographic changes). The divergence K_J‐C_ (Jukes–Cantor correction) was also calculated between the two species and between putative allelic lineages under strong linkage disequilibrium. Linkage disequilibrium among segregating sites and recombination among alleles were tested by calculating the Z_n_S statistics of Kelly (1997) and the minimum number of recombination events (R_m_) of Hudson and Kaplan (1985), respectively. Linked sites were taken into consideration if significant following a Fisher's exact test and a Bonferroni correction. The occurrence of recombinants was also checked using GenConv and Chimaera packages of RDP v3.0 (Heath et al. 2006). The strength and position of a selective event at a given locus was measured by calculating *π*_a_/*π*_s_ (within each species) and K_a_/K_s_ (between species) ratios with DNAsp v5.10 using a sliding window of 50 bp and a step of 10 bp to rule out spurious peaks due to the emergence of nonsynonymous singletons in regions with a low mutation rate. The evolution along the gene of both the divergence and genetic diversity was then examined with a larger sliding window of 100 bp and an extended step of 50 bp in concordance with the position of nonsynonymous mutations fixed in the divergence or found at intermediate frequency in the species polymorphism. Mismatch curves were also produced for all genes and each species using the same software to test whether the distribution of segregating sites follows a model of population expansion. Rogers and Harpending (1992) indeed argued that qualitative and quantitative aspects of a population's genetic history should be uncovered by the analysis of frequency distributions of pairwise sequence mismatches under the hypothesis of no selection. Date at which populations started expansion was estimated with the package Fluctuate of the software LAMARC (Kuhner 2006) according to the exponential growth equation: *θ*_t_ = *θ*_0_ e^−gt^ using a rather high substitution rate of c.a. 2% per My for significant fits to the model of population under expansion.

Phylogenetic trees were constructed from the aligned coding sequences of the alleles with the MEGA 5.0 software using the Minimum Evolution procedure (Kumar et al. 1994). Only the optimal trees for which the sum of branch lengths is minimal are shown. Trees are drawn to scale, with branch lengths in the same units as those of the evolutionary distances used to infer the phylogenetic tree. The evolutionary distances were computed using the p‐distance method which uses the number of base differences per site. The ME tree was searched using the Close‐Neighbor‐Interchange (CNI) algorithm at a search level of 0.

### Statistical tests and departure to neutral evolution

In order to disentangle selective and demographic effects, a series of statistical tests were performed by comparing the number of polymorphic mutations within each species with the number of mutations fixed between the two krill species taking advantage that loci were all coding sequences.

A MacDonald–Kreitman Test (McDonald and Kreitman 1991) was performed using the DnaSP v5.10 software (Librado and Rozas 2009) to test for signatures of selection by comparing the ratio of nonsynonymous and synonymous mutations within each species (*π*_a_/*π*_s_) to the ratio since their divergence (K_a_/K_s_).

### Maximum‐likelihood Sweep_Bott test

This maximum‐likelihood program was used for each species to test the fit of our sequence datasets either to a selective sweep or a bottleneck using all loci including *cox‐1* with a MCMC approach of 100,000 initial iterations, and a second step of 1,000,000 iterations with 20 optimizations processes and a theta prior ranged from 0 to 30. Sweep_Bott computes coalescent trees and fits them against three distinct population models (the null constant size model M1, the bottleneck model M2, and the sweep model M3) using a coalescent‐based likelihood method to determine whether the time and the strength of the diversity reduction is sufficiently similar between loci to suggest an historical bottleneck (M2) or more locus‐dependent to detect a sweep (M3). Selective sweeps (M3) can be inferred by studying departure from the null model for each locus independently since, contrary to expectations in the case of a veritable bottleneck, departure from the neutral model under a selective sweep should affect only a few loci. Because this test assumes an infinite‐site mutation model with no reverse substitution, segregating (homoplasic) sites that did not follow such an assumption were carefully discarded with the Perl script IMgc.pl (Woerner et al. 2007). The best model was selected following a likelihood ratio test between models (LRT).

### Maximum‐likelihood HKA test

A multilocus HKA test (Hudson et al. 1987) was performed separately for both species as it compares both polymorphism and divergence between loci to infer whether some of them depart from neutral evolution. To perform such a test, the MLHKA software was chosen as it conducts maximum‐likelihood analysis of multilocus polymorphism and divergence data for testing for the action of natural selection on candidate genes (Wright and Charlesworth 2004). The likelihood of the null model (M_0_) was evaluated assuming that all loci evolved neutrally (all selection coefficients set to 1) and compared with alternative selective models for which, at least, one locus was allowed to vary under selective constraints (selective coefficient different from 1). The statistical significance of the selective model was assessed by a likelihood ratio test against either the null model or, when more than one locus was under selection, against the one‐locus models that corresponded to the tested loci. Loci with a significant selective coefficient lower than one were considered to have experienced a selective sweep whereas those with a coefficient greater than one to be under balancing selection. A MCMC chain length of 10^6^ was used for each run, and the program was run three times to ensure the stability of results.

## Results

### Gene polymorphism and divergence

Allelic sequences of *hsp70s* (two alleles per individual, one obtained by the MR cloning method and the other deduced from direct sequencing) were used to estimate both genetic diversity and species divergence. Table 2 shows the level of genetic diversity of the four sampled genes including three paralogous loci of the *hsp70* multigene family and a fragment of the mitochondrial *cox‐1* gene in the two closely related species of Antarctic krill. Variation in the number of alleles recovered within each species and gene was mainly due to the recapture success of alleles after cloning. For the three paralogous *hsp70* genes, *E. superba* displayed more segregating sites (S) and a higher nucleotide diversity (as estimated either by *θ*_w_ or *π*, which both gave similar values) than *E. crystallorophias* with at least a tenfold variation between the two levels of polymorphism for both paralogs A and B (see Table 2). To a lesser extent, the nucleotidic diversity was also lower in *E. crystallorophias* at paralog C but remained nearly identical for both species at the *cox‐1* mitochondrial gene. Gene diversity was close to its maximum for all the studied genes (from 92% to 99.6%) for *E. superba*, but was unexpectedly low for *E. crystallorophias* at both paralogs A and B (15% and 56%, respectively). Tajima's D values were slightly negative and not significant for all tests except for the paralog B in *E. superba* for which the value was positive (see Table 2).

**Table 2:** Genetic diversities and associated Tajima's D statistic for each *Hsp70* paralog and the mt*Cox‐1* gene in both species (*Euc: Euphausia crystallorophias*;* Eus*:* Euphausia superba*)

Locus	Species	Number of alleles	Length (bp)	S	*θ*_w_ ± SD	*π *± SD	Hd ± SD	Tajima's D a
Paralog A	*Euc*	26	892	2	0.0006 ± 0.0004	0.0002 ± 0.0001	0.15 ± 0.093	−1.51
Paralog A	*Eus*	26	892	16	0.0047 ± 0.0012	0.0033 ± 0.0005	0.92 ± 0.037	−1.04
Paralog B	*Euc*	18	891	4	0.0013 ± 0.0076	0.0007 ± 0.0002	0.56 ± 0.130	−1.35
Paralog B	*Eus*	24	891	58	0.0180 ± 0.0060	0.0220 ± 0.0010	0.98 ± 0.024	0.85
Paralog C	*Euc*	26	850	40	0.0120 ± 0.0043	0.0110 ± 0.0009	0.99 ± 0.016	−0.38
Paralog C	*Eus*	22	850	53	0.0170 ± 0.0060	0.0120 ± 0.0010	1.00 ± 0.015	−1.12
*cox‐1*	*Euc*	20	558	22	0.0110 ± 0.0043	0.0078 ± 0.0006	0.98 ± 0.024	−1.38
*cox‐1*	*Eus*	23	558	22	0.0110 ± 0.0041	0.0096 ± 0.0007	0.99 ± 0.016	−0.53

a None of the D values were found to be significantly different from zero.

### Coalescent trees

**Figure 2:**
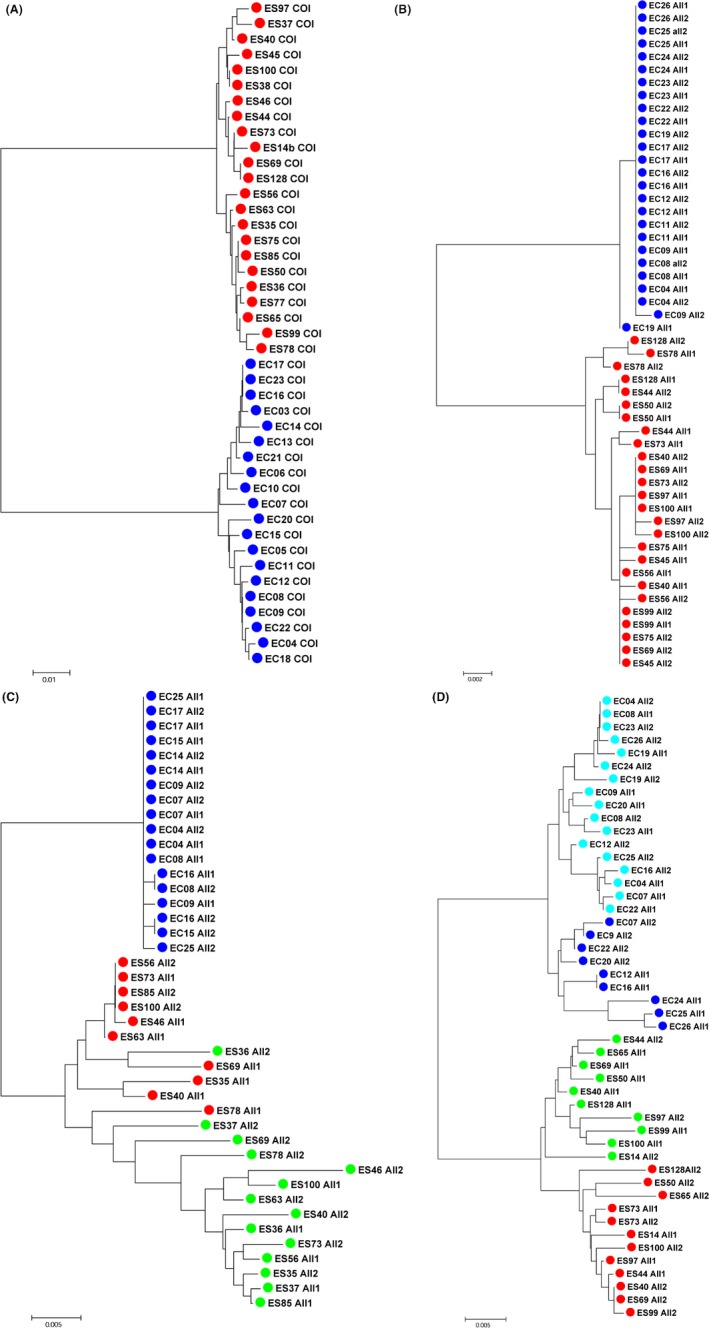
Coalescence tree of (A) *cox‐1*, (B) *hsp70* paralog A, (C) paralog B, (D) paralog C computed with the MEGA 5.0 software using the Minimum Evolution procedure (Kumar et al. 1994). Only the optimal trees for which the sum of branch length is minimal are shown. Trees are drawn to scale, with branch lengths in the same units as those of the evolutionary distances used to infer the phylogenetic tree. The evolutionary distances were computed using the p‐distance method and were in the units of the number of base differences per site. The ME tree was searched using the Close‐Neighbor‐Interchange (CNI) algorithm at a search level of 0. Each color materializes allelic lineages for both species: in paralog B, green color corresponds to the Gly insertion, and the red one to the Gly deletion, in paralog C, the dark (R) and light blue (I) colors correspond to the two R vs I allelic lineages in *E. crystallorophias* whereas the red (K) and green (M) colors represent the two K vs M allelic lineages in *E. superba*. The first two letters (EC or ES) in the sequence label represent the species name and are followed by the individual number and the allele number (All1 or All2).

Minimum evolution‐based phylogenetic trees obtained using Mega 5.0 for the mitochondrial gene and three *hsp70* nuclear genes are presented in Figure 2. The *cox‐1* tree (Fig. 2A) indicated that both species displayed exactly the same patterns of coalescent events with a high level of gene diversity and no departure from neutral evolution (observed vs expected population parameter comparisons by simulating 1000 neutral coalescents using DNAsp v5.10). Polymorphic mutations were all synonymous and randomly distributed among branches suggesting that mutations had accumulated at roughly the same rate in both species since the speciation event, leading to large and nearly similar effective population sizes (*Ne*) between the two species. In contrast, the *hsp70* paralog A (Fig. 2B) displayed a star‐shaped coalescent tree in *E. crystallorophias* due to the predominance of only one allele with very few singletons but followed a more neutral coalescent shape with deeper branched alleles in *E. superba*. The *hsp70* paralog B sequences (Fig. 2C) displayed a pattern of diversification similar to that observed for paralog A. *E. crystallorophias* exhibited the same star‐shaped tree with virtually no allelic variation while *E. superba* displayed a more branch‐shaped coalescent with the co‐occurrence of two divergent allelic lineages differing one from each other by a glycine deletion in residue 102 (GGG ‐> GG) together with other linked nonsynonymous mutations. Most individuals displayed one allele in the first clade and the other in the second one. Three allelic sequences were, however, positioned between these two clades. Using RDP 3.0 and a Bonferroni correction, at least, one of them (*Eus78‐all1*) was a true recombinant with parental types coming from each of the two clades. Finally, the *hsp70* paralog C (Fig. 2D) exhibited the same pattern of allelic diversification in both species with the co‐occurrence of two divergent allelic lineages within each species. In both species, about 80% of the individuals displayed one allele in the first clade and the other in the second clade with a series of recombinants detected across the clades, but none remaining significant after a Bonferroni correction. In both species, allele coalescences were deep branched with long terminal branches, some of which were potential recombinants with unknown sequences (RDP 3.0).

### Evolution of divergence, d_N_/d_S_ ratios, and genetic diversities along genes

#### *cox‐1*

Nucleotidic diversity at the mitochondrial *cox‐1* gene (sequence length = 562 bp and number of sequences = 43) was high (*θ*_W_ = 0.01) with nearly all haplotypes differing one to each other. Substitutions were mostly synonymous with the noticeable exception of five species‐shared polymorphic sites involving the replacement of methionine by isoleucine. Genetic diversities were nearly identical for both species (see Table 2) with a lack of nonsynonymous singletons suggesting strong purifying selection. Divergence of the two species was of about 14.6% with only one type of amino acid replacement (M‐>I). Specific replacements of methionine (ATG) by isoleucine (ATA) codons therefore supported the hypothesis that, in the mitochondrial genome of krill, methionine is encoded by two codons as previously reported for the *Drosophila*, ascidian, and polychaete mitochondrial genomes. The nucleotidic diversity and divergence were both fairly constant along the gene for both species.

#### *hsp70A*

**Figure 3:**
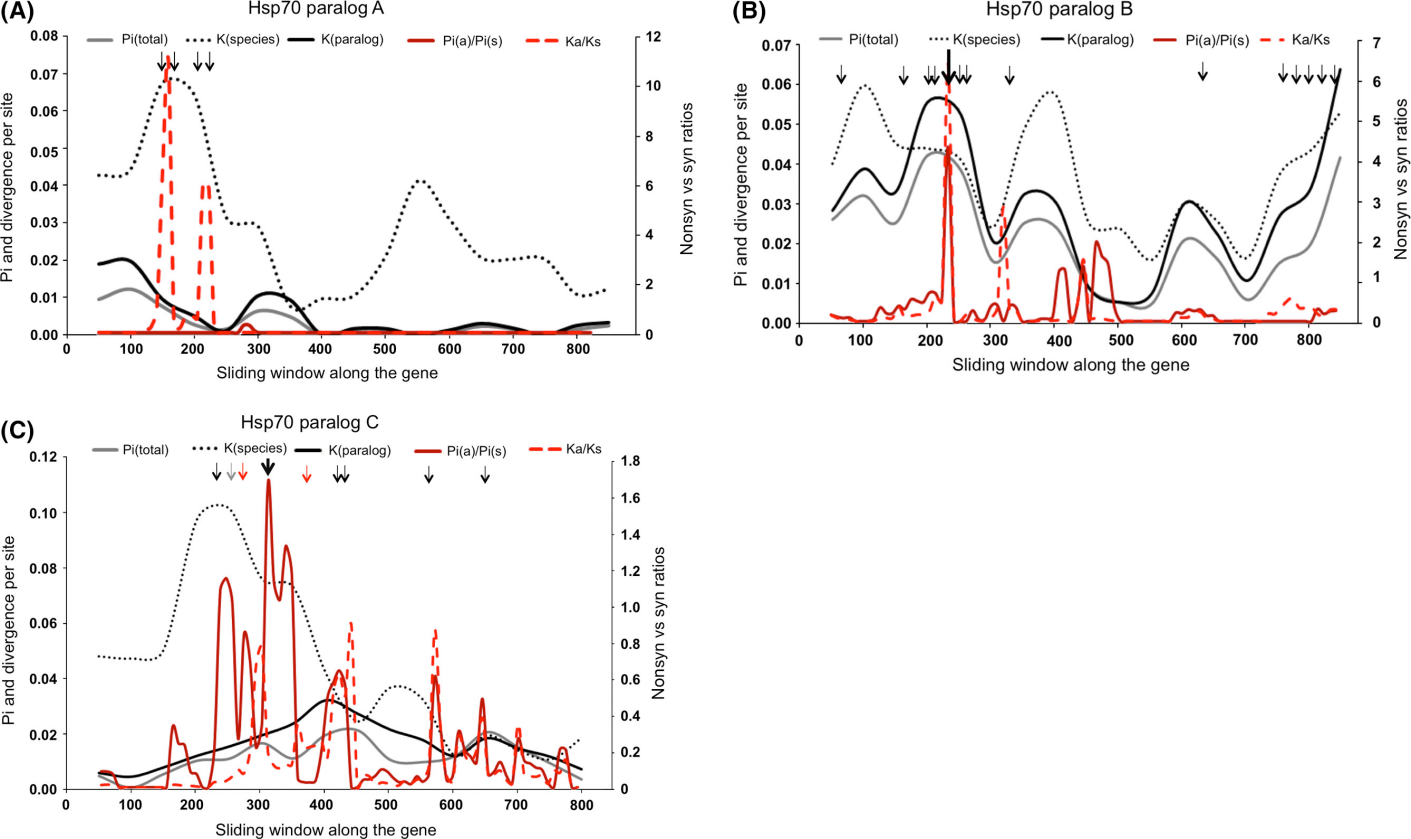
Global genetic diversity (black and gray lines) and species divergence (red lines) along the gene for (A) *hsp70* paralog A. Black arrows indicate the position of nonsynonymous mutations, respectively, IV, DN, SE, TS (B) *hsp70* paralog B. The thin black arrows indicate the position of nonsynonymous mutations between the two Eus allelic lineages (respectively, TS, KN, VI, DS, LI, NT, TA, NT, LF, FL, IV, RK, ED). The bold black arrow indicates the Gly deletion and (C) *hsp70* paralog C. The thin black arrows indicate the position of the nonsynonymous mutations in the *Euc* species (respectively, KN, RS, DK, SC, EV). The thin gray arrow indicates the position of the nonsynonymous mutation KM in the *Eus* species. The bold black arrow indicates the position of the polymorphic hydrophobic/charged site found in the two distinct allelic lineages of the two krill species and, red arrows, the position of nonsynonymous mutations responsible for the divergence of species (respectively, QE and TI). K(species) correspond to the divergence between species and K(paralog) the divergence between allelic lineages within a species for a given paralog.

Examination of sequence polymorphisms over the 892 bp of the *hsp70* paralog A (52 sequences) revealed virtually no genetic diversity in *E. crystallorophias* (*Euc*) with the presence of only one haplotype of 26 allelic sequences. Two distinct lineages co‐occurred in *E. superba* (*Eus*) with strong linkage disequilibrium between at least 5 synonymous segregating sites (Z_n_S = 0.071, 16 significant combinations of 120, but only one remained significant after the Bonferroni procedure). Divergence between the two species was of K_J‐C_ = 0.0282 with the fixation of two compensatory amino acid replacements involving a charged residue (*Eus* ‐> *Euc*: N_63_D and E_74_S), the latter corresponding to a complete codon change (GAA ‐> AGT). Figure 3A showed the evolution of both species and allele divergences together with the overall within‐species nucleotidic diversity (*π*), as well as the *π*_a_/*π*_s_ and K_a_/K_s_ ratios along the gene. The curves clearly positioned the maximum species divergence against both the position of the two fixed nonsynonymous changes and a sharp elevation of the K_a_/K_s_ ratio, suggesting that at least one of these sites could be under positive selection.

#### *hsp70B*

In the specific case of paralog B (sequence length = 892 bp, number of sequences = 42), genetic diversity was strikingly different between the two krill species, with again virtually no genetic variability in *E. crystallorophias* (see Table 2), but with the co‐occurrence of two distinct allelic lineages in *E. superba*. The divergence between the two species (Kspecies) was low (13 changes) with no amino acid replacements between them. By contrast, divergence between the two allelic lineages of *E. superba* (Kparalog) was high with a large number of mutations including 13 amino acid replacements (T_49_S, K_79_N, V_96_I, D_98_S, L_106_I, N_116_T, T_141_A, N_242_T, L_290_F, F_296_L, I_300_V, R_313_K, E_317_D) and the deletion of a glycine residue in one lineage located at position 102. A large number of these segregating sites displayed incomplete linkage disequilibrium with the Gly indel, especially K_79_N, D_98_S, N_116_T, F_296_L, V_300_I, and K_313_R (Z_n_S = 0.139, with 328 significant combinations of 1653 from which 18 remained significant after the Bonferroni procedure). Positions of these amino acid replacements were located on both sides of a sharp peak of *π*_a_/*π*_s_, indicating the accumulation of a great number of nonsynonymous mutations before and after the Gly indel (see Fig. 3B). Average divergences between species (K_J‐C_ = 0.037) and between the two allelic forms of *E. superba* (K_J‐C_ = 0.034) were nearly the same all over the gene (see Fig. 3B). However, one of the two Eus lineages (i.e., containing the *Gly* deletion) was more divergent (K_J‐C_ = 0.042) and diversified (K_J‐C_ = 0.028) from the allelic sequence of *E. crystallorophias*. Moreover, Hudson & Kaplan 4‐point test suggested that several recombinant points existed between the two allelic lineages; however, only one recombinant allele (78‐all1) was formally identified with the MaxChi procedure of RDP 3.0.

#### *hsp70C*

The *hsp70* paralog *C* displayed the highest genetic diversity in both Antarctic krill species (sequence length = 848 bp, number of sequences = 48, Table 2). Both species exhibited two distinct divergent allelic lineages of the same magnitude (*Eus*: K_J‐C_ = 0.016; *Euc* K_J‐C_ = 0.015) with several amino acid replacements (*Eus*: K_86_M, R_170_H; *Euc*: K_76_N, R_139_S, D_140_K S_214_C, and E_247_V), not specifically correlated between the two species. In contrast, although species divergence (K_J‐C_ = 0.043) was much higher than the within‐species allelic divergence, only two amino acid replacements (*Eus* ‐> *Euc*: E_92_Q and I_124_T) were fixed between the two species. Another interesting feature of this paralogous gene was the occurrence of several species‐shared polymorphic sites, including a nonsynomymous one in which two consecutive segregating sites led to the co‐occurrence of L/I (hydrophobic) and R (charged) residues in *E. crystallorophias* and M (hydrophobic) and K (charged) in *E. superba* with intermediate frequencies at position 107. Although the polymorphic residues are different between the two species, the changes affected the nature of the residue in the same way. This polymorphic site was not linked to the two allelic lineages of *E. superba* but displayed a strong linkage disequilibrium with a great number of synomymous sites in *E. crystallorophias* (Z_n_S = 0.101; 80 significant site combinations of 780 with only 4 still significant after the Bonferroni correction). In *E. superba*, linkage disequilibrium was also found at several synonymous sites but without any diagnostic nonsynonymous change (Z_n_S = 0.077; 72 significant combinations of 1378 with 10 still significant after the Bonferroni correction). Both species followed the same patterns of diversity with no recombinant detected between the allelic clades. When looking at the sliding window of species *vs* allele divergence and the *π*_a_/*π*_s_ and K_a_/K_s_ ratios (Fig. 3C), most amino acid replacements were located in the first part of the gene and contributed to the maximum species divergence. This peak of divergence preceded a sharp *π*_a_/*π*_s_ ratio peak and, to a lesser extent, a K_a_/K_s_ peak in the middle of the gene, suggesting that most of the accumulation of nonsynonymous mutations occurred in the vicinity of amino acid changes fixed in either species divergence or between the *E. crystallorophias*’ allelic clades.

### Testing hypotheses of demographic changes

**Figure 4:**
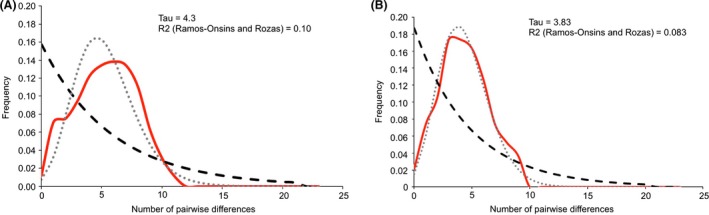
Distributions of the observed pairwise site differences at the *cox‐1* gene (mismatch curves in red line) and the expected values under both the model of growing/declining population (gray dot) and the model of population constant size (black dash) for *E. crystallorophias* (A) and *E. superba* (B) together with their associated Tau and R2 statistics.

Distributions of the frequency of segregating mutations were obtained for the mitochondrial gene *cox‐1* of each species using DNAsp in order to test whether allele coalescences fit a model of demographic expansion under the hypothesis of no selection. Although Tajima's D statistics were not significant, the distribution of mutations fitted, almost perfectly, the theoretical expansion model for both species (Fig. 4). Estimation of the date of expansion led to a period of time of about 400,000 years with the most recent doubling in abundance occurring within the last 100,000 years (Pleistocene). This timing coincides with a polar warming period: the Marine Isotope Stage (MIS) 11 that extended almost 50 kyr (De Vernal and Hillaire‐Marcel 2008), corresponding to the longest interglacial period over the last half million years (Dickson et al. 2009). However, concerning the nuclear genes, results were more heterogeneous (see Fig S1). Because of the lack of mutations at *hsp70* paralog A, the observed mismatch curve was not different from that expected under a demographically stable theoretical model in *E. crystallorophias*. However, with an almost similar mutation pattern, an excess of rare mutations was detected for this species with the *hsp70* paralog B. Conversely, *E. superba* displayed an excess of rare mutations at the paralog A (fitting the population expansion model) but not at paralog B, for which bimodal distribution was observed. Finally, for the *hsp70* paralog C of both species, there was an excess of mutations at intermediate frequencies with bimodal distributions. Single‐locus analyses of nucleotidic polymorphisms therefore provided contrasted information about the putative demographic history of each species in the eastern Antarctic, suggesting that at least, a signal of population expansion was strongly modulated by selection for both species.

To discriminate between these alternative hypotheses, a multilocus approach by maximum likelihood was used with the software Sweep_Bott (Galtier et al. 2000) to fit either a bottleneck or a selective sweep model using our 4 sampling sequence datasets for each species. Model parameters, likelihood and LRTs are summarized in Table 3. For both species, models M2 (population bottleneck) and M3 (sweep) both better explained the gene coalescences than the null model of constant size. However, based on the shape of the gene coalescences, the bottleneck model appeared to better fit for the present patterns of genetic diversity in *E. crystallorophias* whereas the sweep model was more appropriate in *E. superba* (see Table 3).

**Table 3:** Likelihood ratio tests (LRT) performed by the Sweep_Bott software between coalescent models of bottleneck, selective sweeps, or constant population size for each species. 4p, 6p, and 12p represent the number of parameters within each model, namely the four monolocus theta values in M1, the theta values plus the bottleneck strength (S) and the time elapsed since the bottleneck (T) in M2, and the same theta values plus the sweep strength (S_i_) and the time elapsed since the sweep (T_i_) for each locus in M3

Species	Model	Multilocus	Single locus			
		LnL	Paralog A	Paralog B	Paralog C	*cox‐1*
*Eus*	M1: Neutral (4p)	−145.55	−21.46	−39.35	−30.92	−53.81
	M2: Bottleneck (6p)	−138.86	(0.45, 0.97)			
	M3: Selective‐sweep (12p)	−132.15	−16.97	−38.75	−27.1	−49.33
			(0.27, 1.23)	(1.31, 2.88)	(0.28, 1.63)	(0.47, 0.52)
LTR	M2/M1	13.5**				
	M3/M1	26.8***				
	M3/M2	13.4*				
*Euc*	M1: Neutral (4p)	−84.03	−2.88	−5.53	−40.51	−35.1
	M2: Bottleneck (6p)	−4.94	(0.07, 2.1)			
	M3: Selective‐sweep (12p)	−43.55	−0.80	−3.35	−39.39	−0.00
			(0.1, 3.87)	(0.15, 1.49)	(1.00, 0.82)	(0.06, 0.95)
LTR	M2/M1	158.2***				
	M3/M1	80.9***				
	M3/M2	77.2***				

Values in brackets (T, S) correspond to MLE of the time since and the strength of either the bottleneck or the sweep in units of 2N generations, respectively.

Level of significance of the LRT is as follows: *P < 0.05, **P < 0.01, and ***P < 0.001.

### Testing hypotheses of selective sweeps and balancing selection at different loci

#### MacDonald–Kreitman test

To detect whether diversifying selection may have strengthened species divergence or whether balancing selection occurs at a given locus, a MacDonald–Kreitman test was performed on the sequence datasets using nonsynonymous and synonymous mutations accumulated for both the polymorphism within and the divergence between species. Fisher tests had significant *P*‐values for both *hsp70* paralogs B (*P*‐value = 0.0002***, *π*_a_/*π*_s_ = 1.03, K_a_/K_s_ = 0) and C (*P*‐value = 0.0280*, *π*_a_/*π*_s_ = 0.680, K_a_/K_s_ = 0.133) but not paralog A (*P*‐value = 0.700, *π*_a_/*π*_s_ = 0.200, K_a_/K_s_ = 0.330) indicating that selection may have played a role in shaping genetic diversities of heat‐shock proteins by maintaining specific allelic lineages in both species. For paralogs B and C, the *π*_a_/*π*_s_ ratios were indeed much higher than the K_a_/K_s_ ratios allowing conclusions to be drawn about the accumulation of several nonsynonymous mutations at intermediate frequency in the polymorphism of at least one species. In order to confirm whether this excess of nonsynonymous polymorphic mutations could be attributed to an excess of deleterious mutations or, inversely, to the maintenance of advantageous allelic lineages by balancing selection, additional tests were run.

#### Maximum‐likelihood HKA test

The occurrence of either a selective sweep or the maintenance of allelic lineages by balancing selection was tested using the MLHKA software: a multilocus maximum‐likelihood method based on the Hudson–Kreitman–Aguadé test (Wright and Charlesworth 2004). Although the number of loci was low (4), an alternative selective model with only one locus under selection was tested against the neutral model (*Euc*: LnL = −29.45, time since speciation = 2.88; *Eus*: LnL = −28.88, time since speciation = 1.32) for each locus and was powerful enough to detect positive selection (Table 4). This test showed that *hsp70* paralogs A and C were not evolving neutrally in *E. crystallorophias* with selective coefficients of 0.07 (sweep: LnL = −23.97) and 4.17 (balancing selection: LnL = −26.30), respectively (Table 4). This finding was also supported by additional LRT tests comparing a model with two loci under selection with (1) the null model and (2) the retained models for which paralog A or paralog C were under selection. The sequence dataset better fitted a two‐loci model PA&PB (LnL = −22.00) than either the null model or the model with PA alone (LRT = 14.9** and LRT = 3.94*, respectively). Other tests with at least 2 loci under selection were all significantly better than the null model but not when tested against PA or PC alone. With regard to the *E. superba* results, none of the LRTs comparing single‐locus selective models with the null model were significant. However, the model with paralog B and paralog C under selection produced significant LRTs (>4.42*) and a selective coefficient of 3 when compared to either the PB and PC models separately or the null model, reinforcing the hypothesis of balancing selection at, at least, one of these two loci.

**Table 4:** Selective coefficients (k) obtained using the MLHKA test for both species when testing the following model: neutral, one, two, or three locus under positive selection. Maximum‐likelihood scores (LnL) are provided in brackets when significant with reference scores of −29.45 (Euc) and −28.88 (Eus) for the neutral model

Species	One‐locus test				Two and three locus under selection (2‐L&3‐L)			
	*hsp70*A	*hsp70* B	*hsp70* C	*cox‐1*	2‐L (PA&PB)	2‐L (PA&PC)	2‐L(PB&PC)	3‐L
*Euc*								
*hsp70*A (PA)	0.07*** (−23.97)	1	1	1	0.057*** (22.00)	0.12** (22.56)	–	0.095*** (−21.27)
*hsp70* B (PB)	1	0.25	1	1	0.25*** (22.00)	1	–	0.35*** (−21.27)
*hsp70* C (PC)	1	1	4.17* (−26.30)	1	1	3.17** (22.56)	–	2.70*** (−21.27)
*cox‐1*	1	1	1	2.17	1	1	–	1
*Eus*								
*hsp70*A (PA)	0.4	1	1	1	0.53	0.44	1	0.80
*hsp70* B (PB)	1	2.96	1	1	2.98	1	4.45* (−24.96)	4.49
*hsp70* C (PC)	1	1	2.04	1	1	1.76	3.44* (−24.96)	3.23
*cox‐1*	1	1	1	0.45	1	1	1	1

Levels of significance associated with the selective model (M1) when compared to the null model (M0) using a LRT.

*P < 0.05, **P < 0.01, and ***P < 0.01.

### Allele genotyping from genes with distinct allelic clades

In addition to the sequence analysis, *E. superba* was genotyped for the *Gly* deletion in the *hsp70* paralog B in order to test whether the two divergent allelic lineages may represent recent duplicates. All individuals were heterozygotes for the indel, suggesting that a new duplication event arose in *E. superba* after speciation. A fragment of paralog C without indels containing 5 allelic‐diagnostic sites was also genotyped by direct sequencing using the same individuals, as well as the 24 individuals of *E. crystallorophias* to determine their heterozygous/homozygous status. In *E. superba*, the checking of 40 well‐amplified individuals at these diagnostic sites indicated that only two individuals (5%) were clearly homozygous. In *E. crystallorophias*, five individuals (20%) were homozygous at the diagnostic sites, some of which being clearly heterozygous within the same allelic clade over the whole sequence (Fig. 2D) ruling out the hypothesis of duplicated genes. Genotypic classes were significantly different to the Hardy–Weinberg proportions with a great excess in heterozygotes.

## Discussion

Here, we investigated the recent evolution of three *hsp70* paralogs (A, B, C) and the mitochondrial gene (*cox‐1*) in terms of both polymorphism and divergence in two sister species of Euphausiid (*E. superba* and *E. crystallorophias*) since their speciation by vicariance several Mya ago (Patarnello et al. 1996). This was carried out to explore how both the progressive cooling of the Austral Ocean and the recurrence of glacial episodes may have influenced gene diversification in the recent species history of the Antarctic krill, having in mind that, at least one species, *E. superba* is able to encounter greater thermal and hydrostatic pressure variations than its sibling counterpart. The constrained polar environment would have been expected to decrease the number of copies of *hsp70* genes either by selective relaxation on this specific gene family or the loss of some duplicates, consequently leading to the emergence of species with lower plasticity with regard to heat variations.

### Loss of genetic diversity in the ice krill due to a strong and/or recent bottleneck?

Both haplotype and genetic diversities (only synonymous) were very high for the two krill species at the mitochondrial *cox‐1* gene. These results are in accordance with Goodall‐Copestake et al. (2010) who reported that the krill mitochondrial diversity was among the highest when compared to other marine invertebrates (Muths et al. 2009). Moreover, analysis of the mismatch curves showed that the two observed mutation distributions fit almost perfectly with a theoretical expansion model. This agrees with conclusions proposed on the Antarctic krill (*E. superba*) from previous studies carried out with the same mitochondrial marker elsewhere (i.e. Antarctic Peninsula), but also with microsatellites at the scale of the whole Antarctica continent for the same species (Zane et al. 1998; Goodall‐Copestake et al. 2010; Bortolotto et al. 2011). A new information here is that nucleotide diversities and mutation distributions were highly similar between the two krill species, suggesting that they have nearly about the same effective population size. The multilocus analysis of the molecular variation however delivered a more complex and contrasting picture of the species evolution with selection possibly acting differently on *hsp70* genes within each species. From a general point of view, neutrality tests based only on genetic diversity (Tajima's test) showed no significant evidence for selection and/or demographic changes on nuclear genes. However, the Sweep_Bott test, a sophisticated likelihood method that enables discrimination between selective sweeps and bottlenecks based on the shape of coalescent trees, showed that for some of the loci, there was a better fit to the alternative models of bottleneck and/or selective sweeps rather than to the neutral one. Population bottlenecks in krill can be ascribed to environmental change that occurred in the Antarctic ecosystem during glacial episodes. It is well known that both past forced climatic variation and the formation of Antarctic Convergence (i.e., region where Antarctic waters sink beneath the warmer sub‐Antarctic waters) played a pivotal role in driving evolution in the Antarctic biota through population fragmentation, which isolated the Antarctic and sub‐Antarctic species (Clarke et al. 1992; Zane and Patarnello 2000). Hence, there is no doubt that such climatic events could have influenced genetic diversity of endemic species. Generally speaking, sweeps and bottlenecks led to exactly the same patterns of diversity reduction depending on their strength and the timing of their occurrence. However, population bottleneck is likely to affect all loci. When looking at the Sweep_bott results and the shape of coalescent trees, it is quite probable that *E. crystallorophias* has endured a more recent (T = 0.07) or stronger (s = 2.1) bottleneck than *E. superba*. For the coastal ice krill, M2 (bottleneck) was clearly the best model with three loci of 4 exhibiting patterns of reduced diversity. In contrast, demographic changes should have less impacted *E. superba,* which exhibits a greater variance in model parameters across loci and a better fit to the M3 model (LTR M3/M1 = 26.8 *P* < 0.001). The greater strength of bottleneck in the ice krill can be explained by the marked difference in the ecological behavior of the two species (Patarnello et al. 1996; Atkinson et al. 2004, 2008). *E. crystallorophias* is currently found above the continental margin of Antarctica and develops under the ice sheets during the Austral winter.

Although we cannot rule out the fact that *E. crystallorophias* endured a more pronounced bottleneck than its sibling counterpart, another explanation to such a loss of diversity on *hsp70* genes, at least for paralogs A and B which displayed virtually no genetic variation, should be that selective sweeps may have superimposed on the demographic changes. Sweeps may indeed represent an alternative cause for the nucleotidic patterns observed mainly because, if very recent and strong, a bottleneck should also have erased most of the genetic diversity at both the mitochondrial gene and the paralog *hsp70C*. This is obviously not the case for the mitochondrial gene for which gene and nucleotidic diversities of the ice krill are identical to *E. superba*, but also not the case when considering the two allelic lineages of the *hsp70C* gene separately, each allelic clade being 10 times more diversified than each of the two former paralogs. Action of directional selection is also strengthened by the MLHKA test, which displays in *E. crystallorophias* a highly significant sweep signature for paralog A (k = 0.07) and for paralogs A+B (k = 0.06 and k = 0.25). However, because of the very high evolutionary rate associated with the mitochondrial genome, it could be very probable that the accumulation of new mutations occurred more rapidly than in nuclear genes and, thus could explain why the mt *cox‐1* gene was more diversified and close to that of *E. superba* if the bottleneck is old enough. A similar conclusion can also be drawn for the level of genetic diversity found in the *hsp70C* of the ice krill within which the loss of alleles by drift during the bottleneck could have been compensated by the strong balancing selection acting at this locus.

In conclusion, the effect of the Antarctic cyclical instability due to the recurrence of glacial episodes was undoubtedly more severe for *E. crystallorophias*, which had a limited ability to escape the grounding ice advances on the continental shelf and was continuously exposed to subfreezing temperatures (i.e., −1.8°C). In this ecological context, both a population size reduction and a strong directional selection were likely to concur on the loss of genetic diversity. According to the mitochondrial DNA, times since expansion were similar for both species but about ten times greater than those estimated by Bortolotto et al. (2011). This therefore highlights the question of why the coastal ice krill, specifically restricted to the continental shelf, was more sensitive to the initial population bottleneck than its pelagic counterpart, if caused by the same climatic event, 40 to 400 kya. An alternative explanation would be that *E. crystallorophias* was not able to expand at the same rate than its pelagic counterpart simply because migration to offshore waters during the interglacial episode has been prevented by trophic competition between the two species (at least for larval stages): a situation often typifying species forced to expand over their geographic range (Thomas et al. 2001).

### The story of paralog B: gene diversification by adaptive duplication or loss of duplicate by drift?

*hsp70* paralog B displayed both high nucleotidic and haplotypic diversities together with the presence of two monophyletic clades of allelic lineages in *E. superba*. This contrasted markedly with the situation of *E. crystallorophias* for which no genetic variation but an excess of deleterious mutations (i.e., nonsynonymous singletons) occurred. Both the MK and MLHKA tests suggested the action of balancing selection at this paralog for *E. superba* with a marked accumulation of nonsynonymous divergence between the two allelic lineages (13 amino acid replacements and a codon indel of a glycine, Fig. 3B). A RFLP analysis of this gene showed that individuals were always heterozygous for the 2 allelic lineages. Balancing selection often leads to an excess of heterozygotes in the specific case of overdominance (Charlesworth 2006), but 100% heterozygosity is not expected. In this case, the multicopy gene history of *hsp70s* genes and the fact that 100% of individuals are heterozygotes in *E. superba* strongly suggested that the gene had recently duplicated. However, given the results of both the MK and MLHKA tests and the absence of fixed amino acid replacements in the oldest species divergence (K_a_/K_s_ = 0), accumulation of nonsynonymous mutations (13 replacements and the insertion of an additional glycine) between the two “duplicates” cannot be neutral (i.e., selective relaxation following duplication) and strongly suggests that such a duplication should be adaptive. Such a situation may have been triggered by the addition of a third consecutive glycine in a fold of the protein (i.e., the ancestral state is a double glycine when compared to the A paralogs). In addition, this change is polymorphic in *E. superba* while fixed in *E. crystallorophias*. Replacement of large polar residues by a glycine is known to increase the flexibility of the polypeptide backbone (Matthews et al. 1987; Lopez‐Llano et al. 2006) and a subsequent advantage in terms of protein activity in cold habitats (D'Amico et al. 2002; Saunders et al. 2003).

Duplicated genes have been the target of many empirical and theoretical studies since Ohno (1970) who suggested that most genes originated from duplication events, one duplicate being free to evolve from the second that kept the original function of the gene. Since then, numerous genomic studies showed that functional duplicated genes are very common occurrences (Lynch and Conery 2000) and that gene duplication was one of the major forces driving novel functions (Sidow 1996). As such they have played a significant role in the evolution of genomes, in the functional diversification of their products and, ultimately species divergence. Increasing the number of copies of a gene is known to confer a selective advantage notably in term of adaptation to high or low temperatures following recent duplication (Kondrashov 2012; Coppe et al. 2013). Here, the very fast accumulation of nonsynonymous mutations in the divergence of the two paralogous sequences of the gene (at least 7 mutations linked to the *Gly* indel) is highly indicative of allelic specialization for a species able to migrate vertically and thus to encounter different habitats according to depth. Gene diversification by duplication therefore appears to be a strong positive force for the maintenance of “specialized” alleles without any cost in terms of selective mortality and, thus, a case of balancing selection in the early period of the duplication when the paralogous divergence is not strong enough to prevent recombination between duplicates (Innan 2003; Fawcett and Innan 2011).

Alternatively, such a contrasting situation with 2 highly divergent (in terms of nonsynonymous substitutions) duplicates in *E. superba* whereas only one monomorphic allele is observed in *E. crystallorophias* may be also indicative of the secondary loss of one duplicate by drift as a consequence of a strong reduction in size of the population of the latter species. Indeed, *E. crystallorophias* only exhibits the GGG isoform whereas *E. superba* has the two GGG and GG isoforms with the former being greatly less diversified than the latter. Because the GG isoform represents the ancestral form (i.e., present in paralog B for other krill species and in paralogs A for the two Antarctic krill species), it is therefore highly probable that the GGG isoform corresponds to a new mutation that has emerged before speciation. This may also explain why the *Eus* GG lineage (i.e., containing the *Gly* deletion) was more divergent from the *Euc* GGG lineage (K_J‐C_ = 0.042) than both the *Eus* GGG lineage (K_J‐C_ = 0.028) and the species divergence (K_J‐C_ = 0.037). Whatever the likely cause of the diversity difference between the two krill species (the loss of one duplicate in *E. crystallorophias* or the emergence of a new adaptive duplication in *E. superba*), the evolutionary consequence of this past event is that *E. superba* is therefore the only species which possesses the two GG and GGG isoforms in a turn of the protein encoded by paralog B, the GGG form being possibly more flexible than the other: a situation that can be advantageous in cold waters (D'Amico et al. 2002).

### The inducible hsp70C: maintenance of allelic lineages by balancing selection following neofunctionalization

The family of *hsp70s* always displays inducible and constitutive forms in nearly all organisms. In the specific case of the Antarctic and ice krills, five *hsp70* isoforms and a single Grp78 have been described (Clark et al. 2011; Cascella et al. 2015). A few studies have documented the lack of heat‐shock response and more precisely the lack of induction of *hsps* in response to elevated temperatures in a significant proportion of cold‐adapted organisms (Hofmann et al. 2005). In a recent paper, Cascella et al. (2015) examined the thermal induction of *hsp70s* in our 2 krill species and found that the crustacean inducible isoform (i.e., paralog *hsp70C*) seemed to have lost such a property. In the present study, we provide a more detailed study on this specific isoform, which enables us to test more accurately the hypothesis of selective relaxation. Analysis of the coalescent trees showed that both species exhibited the same highly diversified pattern of alleles with two distinct clades within which derived mutations are equally distributed (see: Fig. 2D). At first glance, such a subdivision could have been caused by a recent duplication. However, duplication here is highly improbable for three main reasons. First, given the divergence accumulated between the two species, a duplication preceding speciation is not possible and would have grouped species within each duplicate. Second, if gene duplication arose after speciation, there is very little chance that they would co‐occur independently in the two species at the same time (Faure et al. 2007). Third, if independent species‐specific duplications arose nonetheless, one can expect that derived mutations would differ within each species and would be likely more frequent in one of the two duplicates (one being free to evolve while the other is more prone to keep the original function). Here, derived substitutions are randomly distributed among clades within each species and a non‐negligible proportion of polymorphic sites are shared between species. In order to rule out the possibility of a double duplication, each individual of both species was genotyped at several diagnostic sites to see whether some individuals could be homozygous at one of the two allelic clades. Each species displayed a series of homozygous individuals (about 12% over the two species: also see Fig. 2D), indicating that clades represent true allelic lineages and not duplicated genes. In both species, the divergent alleles accumulated a significant proportion of nonsynonymous mutations that became fixed or nearly fixed in the clade divergence. The MK test showed that an unexpected (non‐neutral) proportion of nonsynonymous replacements were fixed between the two allelic lineages of each species (7 replacements) when compared to the only 2 replacements detected within the 4‐times greater species divergence. Moreover, the presence of a polymorphic nonsynonymous site (charged vs hydrophobic) together with a few adjacent covarions (3 linked codon sites) across the 4 allelic clades suggests that such a polymorphism may have predated speciation. Assuming balancing selection at this site was in good agreement with the excess of heterozygotes (95–80%) depicted by the genotyping. In this specific case, overdominance may act at the maintenance of the different alleles while promoting locally the nonsynonymous allelic divergence on the first part of the gene by preventing recombination between alleles as theoretically predicted by Charlesworth (2006). The particular interest of this site is the dual maintenance of a hydrophobic‐to‐charged change that may impact the structure of the protein in both species. Strengthening the protein folding by increasing hydrophobic forces and the electrostatic bonds is crucial for protein stability and is often relaxed to allow a better flexibility in cold‐adapted proteins (Georlette et al. 2003). Many studies have proven that enzymes from cold‐adapted organisms display structural adaptations to improve flexibility (D'Amico et al. 2002) and even if structural determinants are difficult to elucidate, the analysis of 3D structures often reveals improved accessibility of the catalytic cavity to ligands (Smalas et al. 1994). These observations can easily be extended to the *hsp70C* where balancing selection would have favored the retention of alleles that can be efficient at different thermal regimes (i.e., below and above zero).

In this evident context of cold stability, one should expect that *hsp70*s would evolve under selective relaxation (i.e., d_N_/d_S_ = >1) and ultimately may lose their inducible function as previously shown in other Antarctic species (Hofmann et al. 2000, 2005; Clark and Peck 2009). Based on a phylogenetic reconstruction of the *hsp70* paralogy, Cascella et al. (2015) previously showed that it might be the case as both krill branches of the inducible crustacean isoforms *hsp70 C* were indeed displaying d_N_/d_S_ ratios approaching one. However, present patterns of polymorphism‐to‐divergence at this locus clearly indicated that the two sister species of Antarctic krill were able to maintain allelic lineages characterized by a great nonsynonymous divergence. As a consequence, the apparent loss of inducibility is therefore not the result of a selective relaxation but rather the maintenance of two slightly different protein functions that probably occurred before the separation of the two species. Psychrophiles (i.e., species living at cold/freezing temperatures) display efficient metabolic fluxes at low temperatures that are more or less comparable to those exhibited by closely related mesophiles living at moderate temperatures (Peck et al. 2014). Many studies have tried to understand the biological processes that are involved in the adaptation to cold and especially homeostasis in which *hsp70*s have a crucial role. Here, using a divergence‐to‐polymorphism approach, we propose that the inducible isoforms *hsp70C*, while losing their inducibility, evolved to acquire new unknown homeostasis functions, may be in association with the no‐freezing constraints of the Antarctic fauna. Traces of positive selection seem to be of frequent occurrences in the polar fauna at key specific genes. A genomic study of the polar bear *Ursus maritimus* and brown bear *Ursus arctos* separated 479–343 thousand years ago (Liu et al. 2014), showed that a significant proportion of genes were under positive selection and especially those associated with cardiomyopathy and vascular disease genes (in the polar bear). It appears that bio‐energetic adaptation to life in extremely cold temperatures with periods of fasting also shaped the genetic diversity of polar bears (Welch et al. 2014).

### hsp diversity correlates with the heat resistance of the two krill species

*E. superba* and *E. crystallorophias* both represent emblematic species at the base of the whole trophic web of the Austral Ocean that is reliant upon their evolutionary potential and current population fitness to adapt to increasing temperatures (Spielman et al. 2004). However, although displaying extremely large census sizes (one of the greatest among marine fauna: Goodall‐Copestake et al. 2010), the two krill species displayed strikingly contrasted *hsp70* genetic diversities. While *E. crystallorophias* displayed virtually no genetic diversity at the three *hsp70* loci and is suspected to have lost one of its duplicates at the *hsp70* B gene, *E. superba* appears to be more polymorphic at paralogs B and C with a non‐neutral accumulation of amino acid replacements as evidenced by the MLHKA and MK tests. Either such a situation was dictated by a recent and divergent demographic history (i.e., less than 400 kya) or differential adaptation to specific ecological niches since their speciation (i.e., at least 6 Mya: Patarnello et al. 1996), the heat‐shock protein diversity of the ice krill was more severely impacted. This therefore questions the adaptability of the two krill species in terms of heat fluctuations. To date, there is no general trend between increasing genetic diversity and population fitness. However, according to theoretical prediction, a correlation is expected between the loss of heterozygosity and the population fitness simply because it has been widely documented in inbred species (Booy et al. 2000; Reed and Frankham 2003). Alternatively, selection in heterogeneous environments can also lead to more plastic molecules (both in terms of eurythermal activity and levels of expression) able to cope with a wide range of temperatures rather than a series of specialized alleles (Via et al. 1995; Booy et al. 2000). In the specific case of *hsp70*s, adaptation to thermal changes is not simply a matter of gene diversification but rather a level of plasticity in the expression response of the gene. Previous heat‐shock experiments and physiological analyses of the two krill species allowed us to discriminate between these two alternative scenarios (i.e., positive influence of genetic diversity vs. phenotype plasticity). In this context, *E. crystallorophias*, which appears to be monomorphic at two *hsp70* loci, is also the species which has both the lowest CTmax and the earliest response in terms of *hsp70* induction (see: Table 5, Cascella et al. 2015). It is well admitted to consider that the more a species is heat‐stressed, the more an *hsp70* overexpression is expected in response to the stress. Following this idea, Sorensen et al. (1999, 2001) have shown that thermoresistant *Drosophila* lines have lower levels of *hsp* expression in response to a thermal shock. These results suggest that thermoresistant lines undergo less cell damages and are thus able to produce a lower response: a situation clearly admissible when the two krill species are compared. However, Cascella (2014) also reported that nearly all *hsp70* isoforms had nearly the same limited potential of inducibility in the two Antarctic krill species when compared to their arctico‐boreal relatives, which have, however, similar CTmax values. The proposed hypothesis was that the stock of protein isoforms might be constitutively upregulated as a by‐product of living at temperatures below zero in the Austral Ocean. As a consequence, the difference in the thermal resistance of the two krill species does not only seem to be related to their ability to modulate their levels of *hsp70* expression at a given locus, even if the ice krill is more reactive, but also a matter of biochemical diversification allowing them to explore more cellular pathways (protein refolding, homeostasis, metal sequestration, damaged‐protein elimination, and possibly antifreeze cell protection). Such a positive role of *hsp70* diversification by recurrent duplication events in terms of individual fitness has been well evidenced by Bettencourt and Feder (2001), Bettencourt et al. (1999, 2008) in *Drosophila melanogaster* for which the *hsp* response is directly dependent upon the number of gene copies in the genome.

**Table 5:** Summary of physiological resistance of the Antarctic and Ice krill species to thermal shocks (adapted from Cascella et al. 2015): CTmax and temperature affecting swimming behavior represent the thermal limit at which 50% of the tested individuals have died or have fallen onto the bottom, respectively, following a regular increase of temperature of 0.1°C per minute from an initial temperature of −1°C

Species	Habitat	Depth (m)	Maximum in situ temperature (°C)	CTmax (°C)	Temperature affecting the swimming behavior (°C)	Hsp70 gene expression during thermal shock
*E. superba*	Offshore waters	0–3000	4–5	15.8	8–9	No overexpression at 3 and 6°C for 3 to 6 h
*E. crystallorophias*	Shelf waters	0–500	0–1	14.7	1–2	Overexpression after 3 h at 6°C

### Management perspectives

Adaptation to temperature is a complex process in which many polygenic traits may interfere by epistasis or can be influenced by epigenetic variation (Harrisson et al. 2014). However, using immediate heritable genetic traits to become more plastic may represent a powerful mechanism for surviving environmental changes (Chevin et al. 2010; Reed et al. 2011). Although nothing is known about the level of plasticity of the krill chaperones, our study indicates that *E. crystallorophias* has a less diversified arsenal of *hsp70s* variants, and, according to Cascella et al. (2015), is also physiologically less plastic to thermal shocks. As a consequence, it should be more vulnerable to climatic change when compared to its homolog *E. superba*.

## Conflict of Interest

None declared.

## Data Accessibility

Sequence's GenBank accession numbers for the whole gene are: *EucHsp70A*: KM067139; *EucHsp70B*: KM067140; *EucHsp70C*: KM067142; *EusHsp70A*: KM067144; *EusHsp70B*: KM067145; *EusHsp70C*: KM067147, together with the subsequent allelic forms: accession numbers, *EusHsp70A*:KT586430‐KT586455; *EusHSP70B*:KT586456‐KT586479; *EusHSP70C*:KT586480‐KT586501; *EucHSP70A*:KT586502‐KT586527; *EucHSP70B*:KT586528‐KT586545; *EucHSP70C*: KT586546‐KT586571; *Euscox‐1*: KT586572‐KT586594; *Euccox‐1*: KT586595‐KT586614. The final DNA sequences alignment of each gene can be uploaded as online Supporting Information (Supp1_Krill_COI_fasta.txt. Supp2_Krill_Hsp70A_fasta.txt. Supp3_Krill_Hsp70B_fasta.txt, Supp4_Krill_Hsp70C_fasta.txt). This project (IPEV‐ 1039) was approved by IPEV (Institut Paul Emile Victor, the French Polar Institute) review committee and was declared to and approved by the Terres Australes et Antarctiques Françaises in 2009 according the Annex I of the Madrid Protocol and the French Decret No 2005‐403. No endangered or protected species were used.

## Supporting information


**Fig. S1.** Distributions of observed and expected pairwise site differences at the three Hsp70 loci (mismatch curves) for *E. superba* and *E. crystallorophias*. Black line: observed values, gray line: expected values under the model of constant size and, Gray dot: expected values under the model of growing/declining population.

**Data S1.** Sequence alignment of mtCOI for the two krill species.

**Data S2.** Sequence alignment of Hsp70A for the two krill species.

**Data S3.** Sequence alignment of Hsp70B for the two krill species.

**Data S4.** Sequence alignment of Hsp70C for the two krill species.
