# Supplementary material for: Divergent ecological histories of two sister Antarctic krill species led to contrasted patterns of genetic diversity in their heat‐shock protein (hsp70) arsenal
Source: Ecol Evol. 2016 Feb 9;6(5):1555–75. doi: 10.1002/ece3.1989 (PMC4775515; doi:10.1002/ece3.1989)

Mismatch curves for each hsp 70 locus (A paralog ; B paralog ; C paralog) for the *Euphausia crystallorophias* species. Observed frequencies follow the black line whereas theoretical values are represented by grey lines (dashed lines: constant model; no variation of the population size and continuous line: growth model; population with a growing population size)

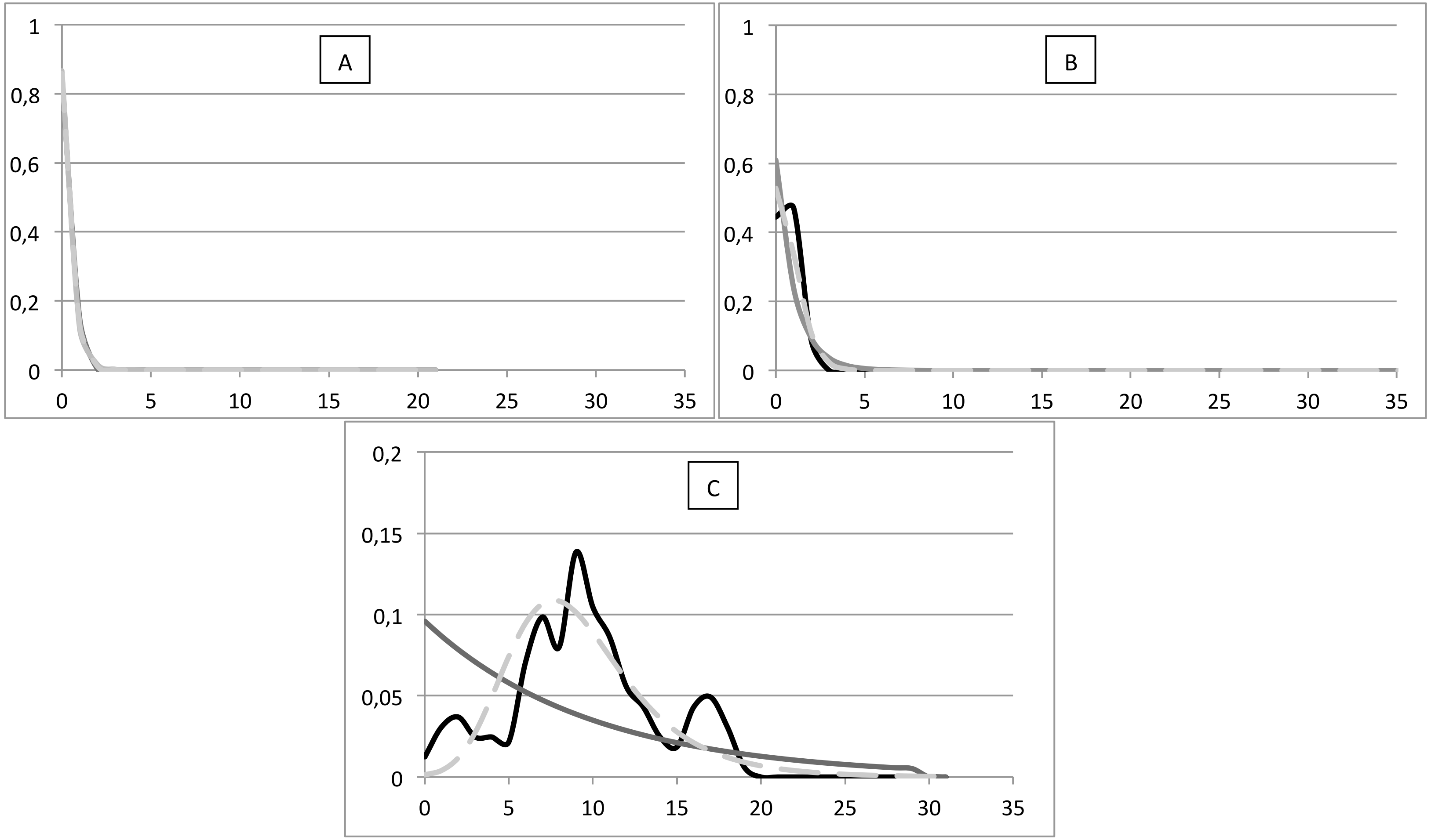

Mismatch curves for each hsp 70 locus (A paralog ; B paralog ; C paralog) for the *Euphausia superba* species. Observed frequencies follow the black line whereas theoretical values are represented by grey lines (dashed lines: constant model; no variation of the population size; continuous line: growth model; population with a growing population size).

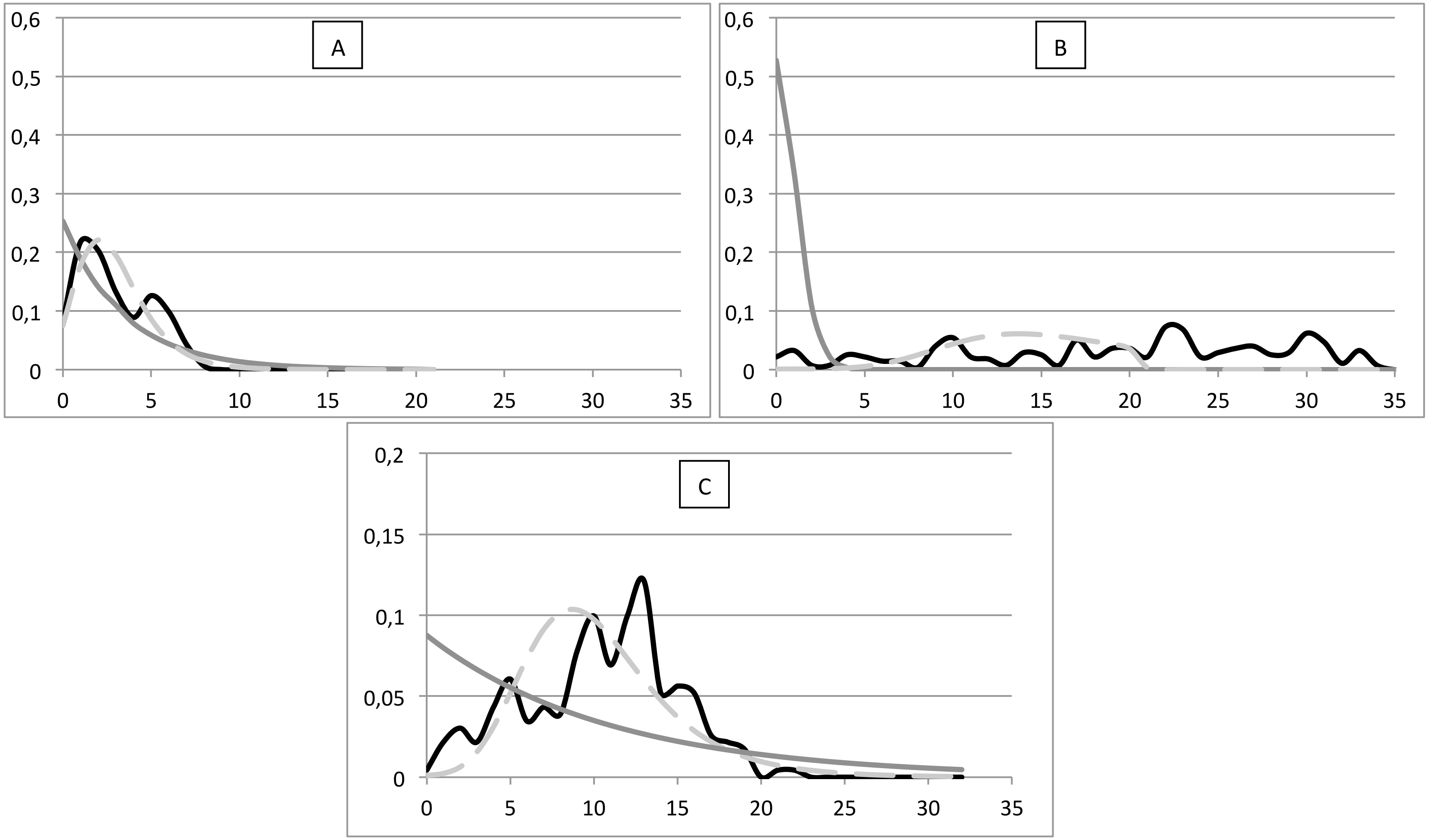

Supplement: Supplementary file 1 — Fig. S1. Distributions of observed and expected pairwise site differences at the three Hsp70 loci (mismatch curves) for E. superba and E. crystallorophias. Black line: observed values, gray line: expected values under the model of constant size and, Gray dot: expected values under the model of growing/declining population. [file ECE3-6-1555-s001.pdf]
